# Is Gastrostomy Justifiable?

**Published:** 1909-02-13

**Authors:** 


### Surgery.

### IS GASTROSTOMY JUSTIFIABLE?

Every case of œsophageal obstruction due to malignant disease presents a difficult problem. Should gastrostomy be performed, and, if so, when?

In the early stages it may not be easy to be certain of the diagnosis. We have to rely largely on the history of the case, the gradually increasing difficulty in swallowing, first solid and then liquid food, leading gradually up to complete obstruction. It is not at all easy to be certain that carcinoma is the cause of the trouble, and there is no known clinical means of making sure that this is the case. On the law of chance the strong probability is that the case is one of carcinoma, because other causes of œsophageal obstruction are exceedingly rare. Stenosis due to syphilis occasionally occurs, and it may follow ulceration after the accidental inhibition of corrosive acids. But in the former case the administration of potassium iodide will clear up the mystery in a short time, while in the latter the history is so definite as to leave no room for doubt.

It might be said that if the case is one of carcinoma, the cachectic condition which is associated with malignant disease will follow sooner or later. But here again we meet with a difficulty, for a patient who is receiving insufficient nutrition on account of an œsophageal stricture, in itself perfectly simple, will lose weight alarmingly, and will soon present all the appearances that we are in the habit of classing together under the name of cachexia.

It is neither a wise nor a helpful proceeding to pass an œsophageal bougie, even if manipulated with the greatest possible care. The veriest tyro in surgery is aware that œsophageal obstruction may be due to an aneurysm of the transverse arch of the aorta, and that to pass a bougie, if this is the case, is to run the risk of causing the patient's sudden death. But even if the obstruction is due to malignant disease, the information which can be obtained from this manœuvre is not enough to counterbalance its danger. At most we can only learn where the obstruction is situated—that is, at which of the three common sites—at the upper end of the gullet or at the cardiac orifice, or at its middle, where it is crossed by the left bronchus; and when we have this information we are no better off because the knowledge thus acquired cannot modify the treatment. Occasionally small fragments of growth are brought up on the end of the bougie, and these can be subjected to microscopical examination and the diagnosis clinched. On the other hand, one hears of cases in which disaster follows. It has been recorded, for instance, that an œsophageal bougie has been passed through the wall of the œsophagus into the peritoneum, and on another occasion into the mediastinum; and again that food introduced by means of a Symonds' tube was found post-mortem in the pleural cavity, the apex of the tube having passed through the wall of the gullet.

The diagnosis must be made therefore largely on the history and by observing the patient. It having been determined that the case is one of carcinoma, should gastrostomy be performed?

In the first place, it must be remembered that a patient may be suffering from carcinoma of the œsophagus and yet not suffer from symptoms of obstruction. His disease will assuredly kill him, but it may do this in quite a different way. It is not at all uncommon, for instance, for the growth to invade the trachea or bronchus and a fatal septic broncho-pneumonia to be set up without any difficulty having been experienced in the ingestion of food. The writer can recall two cases of patients who died from a septic bronchopneumonia, and in whom the real cause, a carcinoma gulæ, was only cleared up at the autopsy. In such a case it is, of course, obvious that gastrostomy could not possibly have served a useful end.

But if symptoms of obstruction supervene, what is to be done? It is certain that the obstruction will become progressively more acute until a stage is reached when gastrostomy becomes imperative if the patient is to be saved from death by actual starvation. In our opinion, it is reasonable to delay the operation as long as the patient can still obtain sufficient nourishment *per vias naturales*. This is best judged by keeping a record of his weight. There is no need to operate as long as the weight is maintained.

As in all cases of organic obstruction of a tube in the body, there is superadded a certain element of spasm; and partial relief can be obtained by means of anti-spasmodics, particularly belladonna and cocaine. Another thing to be borne in mind is the fact that patients may continue to keep up their strength when properly nursed and fed, but that if the supervision is relaxed and they are left to their own devices they speedily begin to go downhill; this is especially the case with hospital patients.

If, in spite of treatment, the patient begins to lose weight and he experiences a progressively greater difficulty in swallowing, operation should not be longer delayed, although it must be confessed that the results are not encouraging, for the operation itself is often just sufficient to upset the patient's equilibrium. Why this is so it is difficult to say, for, as abdominal operations go, it is an easy proceeding, takes very little time, and is not associated with shock. But the fact remains that it often determines the onset of heart failure or bronchitis.

It would also appear that the growth often increases at an accelerated rate after the operation. In colostomy the passage of irritants over the surface of the rectal growth is almost entirely prevented. But this is not the case in gastrostomy, for, although no food now passes along the œsophagus, mucus and saliva still does so, and this is sufficient to keep up the irritation. The duration of life is therefore very short after this operation when it is done for carcinoma. Satisfactory results are only obtained when the diagnosis has been faulty and the condition is subsequently found to be an innocent one.

